# Objective Extraction of Evoked Event-Related Oscillation from Time-Frequency Representation of Event-Related Potentials

**DOI:** 10.1155/2020/8841354

**Published:** 2020-12-19

**Authors:** Guanghui Zhang, Xueyan Li, Fengyu Cong

**Affiliations:** ^1^School of Biomedical Engineering, Faculty of Electronic Information and Electrical Engineering, Dalian University of Technology, Dalian 116024, China; ^2^Faculty of Information Technology, University of Jyväskylä, Jyväskylä 40014, Finland; ^3^School of Foreign Languages, Dalian University of Technology, Dalian 116024, China; ^4^School of Artificial Intelligence, Faculty of Electronic Information and Electrical Engineering, Dalian University of Technology, Dalian 116024, China; ^5^Key Laboratory of Integrated Circuit and Biomedical Electronic System, Dalian University of Technology, Dalian 116024, China

## Abstract

Evoked event-related oscillations (EROs) have been widely used to explore the mechanisms of brain activities for both normal people and neuropsychiatric disease patients. In most previous studies, the calculation of the regions of evoked EROs of interest is commonly based on a predefined time window and a frequency range given by the experimenter, which tends to be subjective. Additionally, evoked EROs sometimes cannot be fully extracted using the conventional time-frequency analysis (TFA) because they may be overlapped with each other or with artifacts in time, frequency, and space domains. To further investigate the related neuronal processes, a novel approach was proposed including three steps: (1) extract the temporal and spatial components of interest simultaneously by temporal principal component analysis (PCA) and Promax rotation and project them to the electrode fields for correcting their variance and polarity indeterminacies, (2) calculate the time-frequency representations (TFRs) of the back-projected components, and (3) compute the regions of evoked EROs of interest on TFRs objectively using the edge detection algorithm. We performed this novel approach, conventional TFA, and TFA-PCA to analyse both the synthetic datasets with different levels of SNR and an actual ERP dataset in a two-factor paradigm of waiting time (short/long) and feedback (loss/gain) separately. Synthetic datasets results indicated that N2-theta and P3-delta oscillations can be stably detected from different SNR-simulated datasets using the proposed approach, but, by comparison, only one oscillation was obtained via the last two approaches. Furthermore, regarding the actual dataset, the statistical results for the proposed approach revealed that P3-delta was sensitive to the waiting time but not for that of the other approaches. This study manifested that the proposed approach could objectively extract evoked EROs of interest, which allows a better understanding of the modulations of the oscillatory responses.

## 1. Introduction

EEG has been widely used in neuroscience field to evaluate the temporal, spectral, and spatial dynamics of cognitive processes. One typical technique is event-related potential (ERP), which is obtained by averaging multitrial EEG data, and the other one is evoked event-related oscillation (ERO) in the time, frequency, or time-frequency domains based on the ERPs [1]. Evoked EROs have been applied for investigating the distinctions of cognitive functions between normal and neuropsychiatric disordered people [2, 3], and different approaches can be employed to obtain evoked EROs, such as digital filtering (like 4-8 Hz for theta band), power spectral density-based spectral analysis, and time-frequency analysis (TFA) [4]. It should be noted that the underlying ideas of calculating evoked EROs by the first two approaches are similar, and the amplitudes are measured either in the time or frequency domains. In terms of the digital filtering method, evoked EROs are obtained by filtering the ERP waveforms (i.e., the averaged EEG data over signal trials) with a band-pass filter, and then, the power of the filtered signals is analysed in the time domain. However, it is difficult to see how evoked EROs change with frequencies in each time point. The approach of TFA can overcome this obstacle, allowing the examination of evoked EROs both in time and frequency domains simultaneously.

Nevertheless, TFA also has its drawbacks in exploring evoked EROs of interest in multicondition ERP experiments. In most previous investigations, the power of evoked EROs was usually calculated in a predefined region with a particular time window and a frequency range. This predefined region was commonly settled down based on the visual inspection of grand averaged time-frequency representation (TFR) distributions in computing the related energies [5–11] and was conventionally computed in a rectangle region so that the method was named as “conventional rectangle method.” However, the shape of evoked EROs, in reality, was more like a waterdrop than a rectangle. If the predefined rectangle region was smaller than the real waterdrop shape of evoked EROs, some useful information would be neglected. Similarly, when the predefined region was larger than the real boundary of evoked EROs, unrelated information would be involved. As reported in these studies [5, 7, 12], the other drawback should also be considered that the number of the visible evoked EROs identified from the grand averaged TFR was smaller than the number of the practical analysed ERPs. Thus, it remains challenges that the expatiations of some stages of cognitive processes would not be present. Bernat et al. [13, 14] suggested that those EROs, which were overlapped in the time and frequency domains, could be effectively extracted by performing principal component analysis (PCA) and Varimax rotation on the matrix of TFRs (i.e., time and frequency domains were rearranged into columns, and the other variables, such as channels, conditions, and subjects, were integrated into rows) in a multicondition ERP experiment (we called this method as “TFA-PCA” here). One of his studies revealed that the decomposed delta and theta oscillations by TFA-PCA were greatly associated with the N2-P3 [15], whereas they merely explained the occurred time course of the selected theta or delta was closest to that of the N2-P3 complex and did not demonstrate which ERP made the most contributions to theta or delta oscillations. Importantly, the core idea of TFA-PCA was to weight the extracted components with the original TFRs, which would result in the decomposed EROs might be still mixtures.

**Figure 1:**
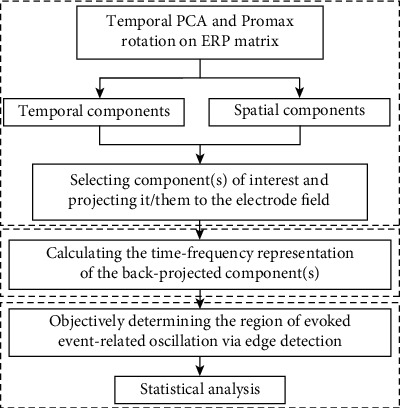
The diagram for extracting evoked event-related oscillations (EROs) from ERP datasets using the proposed method. First, exploring the temporal and spatial components of interest using temporal principal component analysis (t-PCA) and Promax rotation and projecting them to the electrode fields. Second, transforming the projection of the components of interest into time-frequency representations (TFRs) using complex morlet continuous wavelet transform. Third, determining the time and frequency positions of evoked event-related oscillation objectively using edge detection technique for statistical analysis.

To address these gaps, we proposed an approach to objectively extract evoked EROs of interest (the illustration of the proposed approach was displayed in Figure 1). More specifically, temporal PCA (t-PCA) and Promax rotation were conducted to extract the temporal and spatial components. Afterward, the components of interest were selected and projected to the electrode fields for correcting the variance and polarity indeterminacies. It was noted that the back-projection procedure was also used to tackle the problem that several components could not be analysed together in the previous PCA toolbox, like Dien's PCA toolbox [16]. Next, a complex morlet continuous wavelet transform was applied to compute the TFRs of the back-projected component(s) in the electrode fields. Finally, an edge detection algorithm based on Canny detector was introduced to calculate the specific time and frequency positions of evoked EROs from the associated TFRs for further statistical analysis. In addition, correlation coefficients between the topographies of any two participants were calculated to evaluate the homogeneity of ERPs/components/evoked EROs.

In order to evaluate the results of the proposed approach and the other existing approaches, the proposed approach, the conventional TFA, and TFA-PCA were performed on the simulation datasets which were contaminated by different levels of noise (i.e., 20 dB, 10 dB, 5 dB, and 1 dB). As a result, we could obtain the stably results from those simulation datasets using our proposed approach. Meanwhile, the results for the datasets with different levels of SNR, all the extracted components in the time-space domain, and the associated TFRs of evoked EROs in the time-frequency domain were much closer to their sources. We demonstrated this supposition with two aspects as below. One aspect, for different levels of noise-contaminated simulation datasets, we separately calculated the correlation coefficients between any two of the waveforms/topographies/TFRs of the source, mixed, and extracted signals; We also computed the correlation coefficients between the TFRs of the source signal and weighted TFRs obtained by TFA-PCA. The other aspect was to illustrate TFRs obtained by the conventional TFA, the proposed approach, and TFA-PCA. The waveforms/topographies of the source, mixed, and extracted signals were also displayed when SNR is equal to 10 dB. Meanwhile, we also, respectively, performed the proposed, TFA, and TFA-PCA approaches on a real ERP dataset to extract evoked EROs of interest.

In this study, we used the notation of “component(s)” to represent the results obtained by t-PCA and Promax rotation. Likewise, the results gained by the back-projection procedure were considered to be “projected N2/P3”; N2 and P3 were labelled as “ERP” in the original signals; the time-frequency results computed by the conventional TFA, the proposed approach, and TFA-PCA were, respectively, named as “TFR,” “extracted TFR,” and “weighted TFR.” The related codes for the proposed approach can be found from this link: https://guanghuizhang0328.github.io/publications/.

## 2. Data Collections and Methods

### 2.1. EEG and Synthetic Dataset Collection and Analysis

#### 2.1.1. Synthetic Dataset

**Figure 2:**
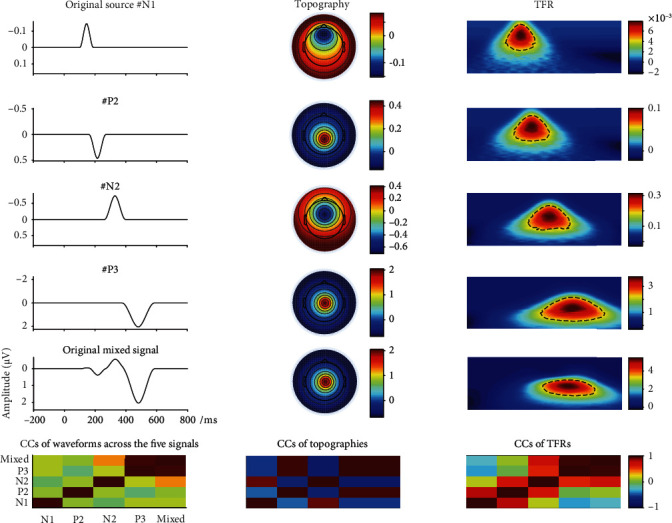
The waveforms, associated topographies, and time-frequency representations (TFRs) for single source N1 (Fz), P2 (CPz), N2 (FCz), P3 (Cz), and mixed signal (Cz), respectively (the first five rows). The last row represents the correlation coefficients (CCs) of waveforms/topographies/TFRs among all five signals. The 65 sets of simulation signals were generated from these sources based on setting the variations of amplitude and latency for N2/P3.

**Figure 3:**
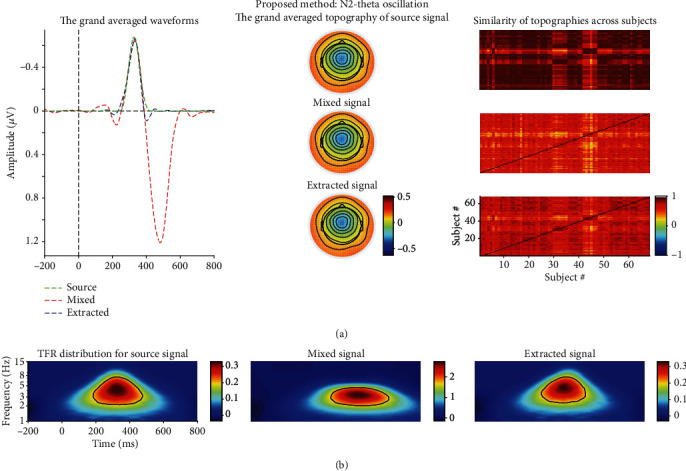
(a) The grand averaged waveform at FCz electrode, topography (the time window was from 300 to 400 ms), and similarity of topographies among subjects of the source/mixed/extracted N2 for the synthetic dataset when SNR is equal to 10 dB. (b) The associated grand averaged time-frequency representations (TFRs) at FCz of the source, mixed, and extracted (using the proposed approach) signals for N2-theta oscillation separately. The mixed signal plays the role of the preprocessed ERPs with the consideration of a real ERP dataset.

**Figure 4:**
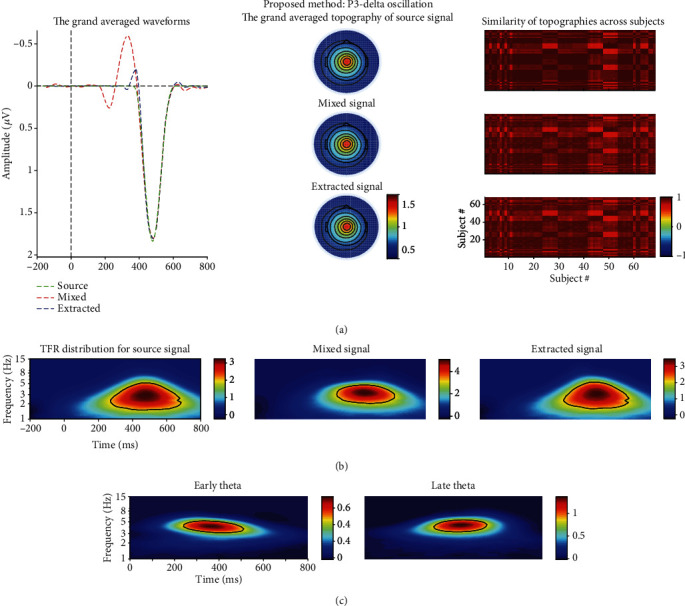
(a) The grand averaged waveform at Cz electrode, topography (the time window was from 400 to 550 ms), and similarity of topographies among subjects of the source/mixed/extracted P3 for the synthetic dataset when SNR is equal to 10 dB. (b) The associated grand averaged time-frequency representations (TFRs) at Cz of the source, mixed, and extracted (by the proposed approach) signals for P3-delta oscillation separately. The mixed signal plays the role of the preprocessed ERPs with the consideration of a real ERP dataset. (c) Weighted TFRs of early and late theta using TFA-PCA. The mixed signal plays the role of the preprocessed ERPs with the consideration of a real ERP dataset.

The synthetic signal was generated with “Dipole-Simulator” (BESA Tool version; it can be downloaded from: http://www.besa.de/updates/tools). The duration of the signal was 1000 ms (from -200 ms to 800 ms). The sampling rate was 150 Hz. There were four simulated ERPs (N1, P2, N2, and P3) whose maximum amplitudes were measured at electrodes Fz, CPz, FCz, and Cz, respectively. In this study, N2 and P3 were considered as the interested ERPs and others were deemed concomitant ones. The maximum negative peaks for N2 and P3 were located at 260-400 ms and 370-580 ms, separately. The details of their associated waveforms, topographic maps in the time domain, and TFR distributions could be found in Figure 2. Meanwhile, we also displayed correlation coefficients between any two of waveforms/topographic maps/TFRs of the four original sources and their mixture to show the degree of overlap and how much the four original sources contribute to the original mixed signal (see the last row in Figure 2). In order to simulate the signals as close to the actual ERP signals as possible, the variations were set in latency and amplitude of P3 and N2 of the original mixed signal (as illustrated in Figure 2), which was applied to simulate the single trial dataset [17]. Following this idea, the 68-set data were subsequently simulated. Different levels of white Gaussian noise were, respectively, added to the mixed 68-set signals (as shown in Figures 3 and 4; the filtered mixed signal plays the role of a real preprocessed ERP dataset), and the signal-noise-ratio (SNR) was set to 20 dB, 10 dB, 5 dB, and 1 dB separately.

#### 2.1.2. Actual Dataset

Twenty-one undergraduate and graduate students were recruited to participate as paid volunteers in the collection of the actual dataset. Nine were females and twelve were males (mean age: 20.95 years old). All the subjects were right-handed, with normal or corrected to normal visual acuity, and they did not know or see the experimental paradigm before the experiment. The details of the experiment materials and the paradigm can be found in this research [18]. EEG recordings at 64 locations were collected according to the standard 10-20 system (Brain Products GmbH, Gilching, Germany). The EEG data were referenced online against the left and right mastoids. Meanwhile, we also collected the vertical and horizontal electrooculogram (EOG) from four electrodes which were placed above and below the right eye and on the outer canthus of the right and left eyes, respectively. All impedances were less than 10 k*Ω* for each electrode. The EEG and EOG for each participant were recorded with a 500 Hz sampling rate, and the data were filtered between 0.01 and 100 Hz using a band-pass filter. The signals from six electrodes (i.e., “HEOL,” “VEOD,” “HEOR,” “VEOU,” “M1,” and “M2”) were not involved in further analysis.

#### 2.1.3. Data Preprocessing and Analysis

*(1) Synthetic Dataset*. According to our previous study [19], as for the frequency band of the components of interest, the synthetic datasets with different levels of SNR were first filtered, respectively, using wavelet filter with the following parameters: the number of levels for decomposition was 8; the selected mother wavelet was “rbio6.8”; the detail coefficients of the number of levels at 4, 5, 6, 7, and 8 were chosen for signal reconstruction. Temporal PCA and Promax rotation were then employed to extract the components of interest and project them to the electrode fields for correcting their variance and polarity indeterminacies. Sequentially, TFRs were calculated by the wavelet transform for the source, mixed, and projected signals separately. During this step, aiming at obtaining better time resolution and frequency resolution of TFRs, the centre frequency and bandwidth were set as 1, respectively, to define a mother wavelet as applied in our previous study [20]. The frequency range of interest was defined from 1 to 15 Hz with 30 frequency bins in nonlinear distribution. For each frequency layer, the power values were baseline corrected by subtracting the mean power of the baseline (200 ms before the stimulus onset) for each point using the subtraction approach [21–23].

We also examined the noise-contaminated simulation datasets by performing PCA on the matrix of TFRs of the mixed signal with 4420 cases (65 channels by 68 subjects) and 3600 variables (30 frequency bins by 120 time point, that is, frequencies ranging from 1 to 15 Hz and time ranging from 0 to 800 ms) using covariance matrix with Kaiser normalization and Varimax rotation [13, 14, 24]. Then, we selected the components of interest from the separated ones and weighted them with the original TFRs based on the main functions of the Bernat's toolbox (http://www.ccnlab.umd.edu/Psychophysiology_Toolbox).

**Figure 5:**
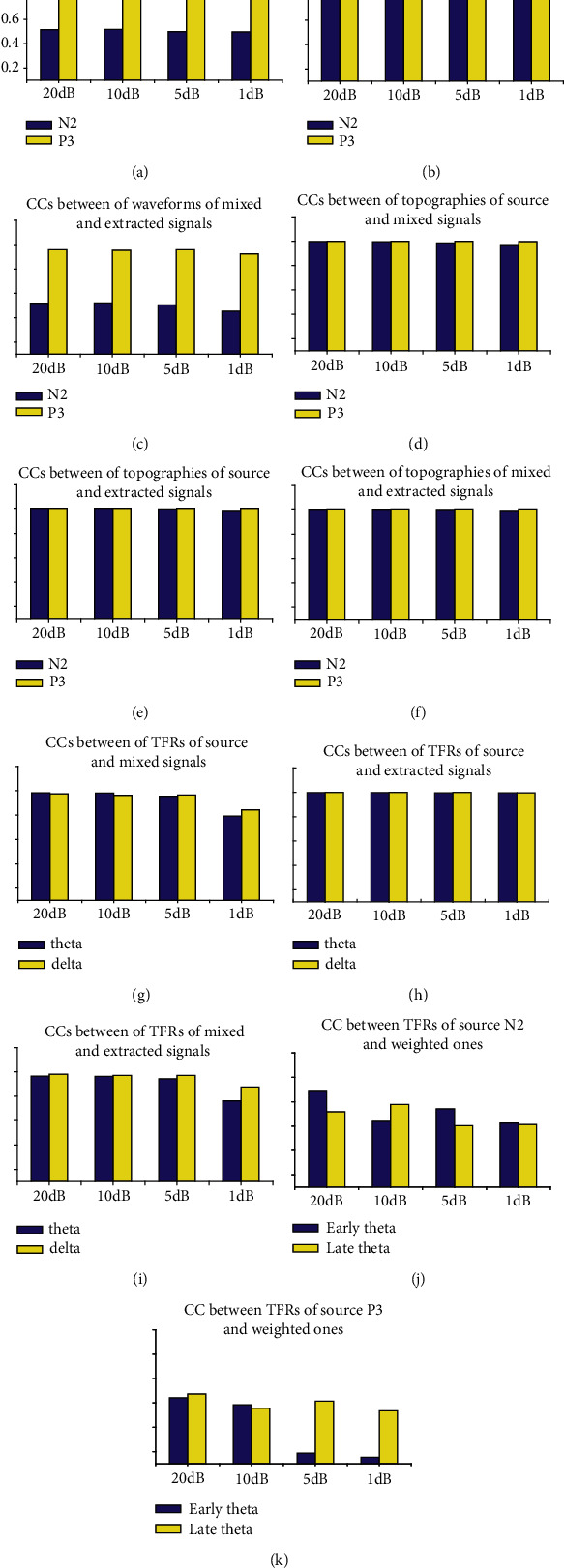
(a–i) The correlation coefficients (CCs) between any two waveforms/topographies/time-frequency representations (TFRs) of the source, mixed, and proposed method extracted N2 (theta)/P3 (delta) for the synthetic datasets with different levels of SNR (i.e., 20 dB, 10 dB, 5 dB, and 1 dB), respectively. (j, k) The CCs between the weighted TFR of source N2/P3 and early/late theta oscillations (using TFA-PCA method), respectively.

To verify that the proposed approach could efficiently extract the evoked EROs of interest from the noise contaminated with different SNR levels without changing their TFR properties, the correlation coefficients between any two of the waveforms/topographies/TFRs of the source, mixed, and extracted signals were separately computed as illustrated in Figures 5(a)–5(i). Likewise, the correlation coefficients between the weighted TFRs of early/late theta and source N2/P3 were also measured (see Figures 5(j) and 5(k)). Furthermore, the related waveforms/topographies in the time domain and TFRs were also plotted for the source and mixed signals (see Figures 3 and 4) when SNR was set to 10 dB.

*(2) Actual Dataset*. The actual datasets were first resampled to 128 Hz so that PCA and Varimax rotation could be performed on the TFRs of the averaged signal with the comparable sampling rate to the simulation datasets. The EEG signals were then filtered offline using a notch FIR filter with 45-55 Hz and a low pass FIR filter with 30 Hz. Sequentially, the filtered continuous recordings were segmented from 200 ms before the stimulus onset to 1000 ms after the stimulus onset. Epochs whose magnitude exceeded ±100 *μ*V were excluded (6.93% epochs were rejected), and the remaining ones were baseline corrected. Next, the multitrial datasets were averaged across every condition of each participant, and the averaged datasets were then filtered by the wavelet filter as used above to improve the SNR.

In order to extract the evoked EROs by the proposed approach, temporal PCA and Promax rotation were performed on the filtered signals to obtain the components related to N2/P3 and project them to all electrodes. To obtain the TFRs of the original averaged and projected signals separately, the frequency range of interest was then set from 0.5 to 14.5 Hz with 30 frequency bins. Additionally, the centre frequency and bandwidth were also set as 1, respectively, as used above for the noise-contaminated simulation datasets.

Another comparison method was also applied to extract the delta and theta oscillatory responses from the TFRs (obtained from the averaged ERP signals) with a frequency range of 0.5-14.5 Hz and time window of 0-1000 ms. Namely, PCA and Varimax rotation were first performed on the matrix formed by TFRs of the original filtered signal with 4872 cases (58 channels by 4 conditions by 21 subjects) and 3840 variables (30 frequency bins by 128 time points). Sequentially, the weighting procedure was separately achieved between the original TFR and the selected components.

The conventional rectangle method and edge detection algorithm were, respectively, conducted to obtain the region of ERO from the TFR of each condition for the conventional TFA (“M1”), the proposed approach (“M2”), and TFA-PCA (“M3”). The power of theta oscillation for each condition was measured in the averaged TFR at Fz, FCz, and Cz electrodes, and the delta oscillation energy was computed at five electrodes Fz, FCz, Cz, CPz, and Pz.

Briefly, with regard to the theta oscillation, when the conventional rectangle method was applied to determine the regions of the oscillatory responses, two regions were measured (“R1”: 100-300 ms and 3-7 Hz; “R2”: 200-400mms and 3-7 Hz) from the grand averaged TFRs. We also, respectively, predefined “R3” (4-8 Hz and 150-300 ms) and “R5” (100-400 ms and 3-7 Hz) in the TFRs of the proposed approach and TFA-PCA to compute the related energies. In addition, the determined regions of the evoked EROs for the last two methods were named as “R4” and “R6” when using the edge detection algorithm. The statistical results were not computed for delta oscillation of the conventional TFA because we did not find the region using the edge detection algorithm.

In terms of delta oscillation, using conventional rectangle method, we also calculated two regions of every condition of TFR obtained by the conventional TFA (“R7”: 200-600 ms and 0.5-2 Hz; “R8”: 300-600 ms and 0.5-2 Hz). Likewise, we used “R10” (1-3 Hz and 200-600 ms) and “R12” (200-600 ms and 0.5-2 Hz) to calculate the power of delta oscillation obtained by the proposed approach and TFA-PCA, respectively. The recognized regions of delta oscillations using the edge detection algorithm for the conventional TFA, the proposed approach, and TFA-PCA corresponded to “R9,” “R11,” and “R13,” respectively.

Finally, two-way repeated-measurement-ANOVA (rm-ANOVA) with waiting time (short/long) and feedback valence (loss/gain) as within-subject factors was used for analysing each determined region of delta and theta oscillations separately. The correction of the number of degrees of freedom would be carried out by the Greenhouse-Geisser method if necessary. All displayed topographic maps in the time domain for simulation and real datasets were obtained using the mean values of the predefined time window. Meanwhile, during PCA procedure, the singular value decomposition was used to decompose the original matrix formed by ERP signals into the sum of several principal components using Matlab function-pca with default parameters (version 2018b, the Mathworks, Inc., Natick, MA).

### 2.2. Proposed Approach for Data Processing

In order to overcome the challenges that evoked EROs could not be extracted completely by the conventional TFA or TFA-PCA approaches, we used the following steps to extract evoked EROs of interest. Firstly, a matrix $\hat{Z}=Z^{T}\in {ℛ}^{N\times M}$was separately formed from the synthetic datasets with different noise levels and real datasets separately to explore the component(s) of interest [25–28]. Herein, it should be noted that time samples were variables in columns of matrix $\hat{Z}$, and the other factors, such as channels, conditions, and subjects, were integrated into rows that were labelled as observations. Then, t-PCA and Promax rotation were fulfilled to decompose this matrix into *R* components, and the components of interest were selected to project to all of the scalp electrodes for correcting the variance and polarity indeterminacies. Subsequently, the calculation of the TFRs of the back-projected components was carried out at all electrodes. Finally, the determination of the regions of evoked EROs at the typical electrodes was worked out using the edge detection algorithm.

#### 2.2.1. Extracting the Components of Interest and Their Back-Projection

The purpose of the t-PCA and Promax rotation was to use a smaller set of nonredundant descriptive variables (i.e., components) to represent the original ERP signal $\hat{Z}$ and then choose the interested components for back-projection (see *Appendix*A and *Appendix*B for the details of the related theories). Importantly, four steps needed to be done during this procedure as below.

The first was about the determination of the number of the remained principal components (PCs). The number of the remained PCs was usually determined based on a predefined percentage ratio, such as 95% or 99%. Such a regulation has been widely applied in various fields. The calculation of this percentage ration was achieved by the sum of a certain number of lambda values over the sum of all lambda values (i.e., *L* = ∑_*r*=1_^*R*^*λ*_*r*_/∑_*m*=1_^*M*^*λ*_*m*_, where *R* is the number of the retained PCs; *M* is the number of the columns of the matrix $\hat{Z}$, *M* > *R*; this percentage ratio was named as cumulative explained variance here) [29, 30].

The second was about the selection of the rotation method. Promax rotation could generate better results than Varimax rotation [31], and it was more efficient for t-PCA decomposition [32]. Hence, Promax rotation was also applied to the study.

The third was about the selection of the temporal and spatial components of interest. If the temporal and spatial properties of the extracted components were consistent with the interested ERPs and its correlation coefficients between any two spatial components of subjects were higher (for example, more than 0.4), the components were then considered for the next analysis. Overall, in terms of the following three aspects, the projected components for ERPs of interest were selected [25]: (a) the polarity and latency of temporal component; (b) the polarity and location of the excitation region of spatial component; (c) the correlation coefficients between any two spatial components, herein spatial components were topographies, of every condition.

The fourth was about the back-projection ${\left(\underline{Z}\right)}^{T}$ of the selected components to the electrode fields. The components, derived from blind separation algorithm [33], herein t-PCA and Promax rotation, had the polarity and the variance indeterminacies, and the back-projection theory could be applied to correct them [34–37]. In practice, ERPs were often decomposed into several temporal and spatial components due to the fluctuation of the original waveforms of the interested ERPs over different subjects. Thus, all of them should be selected to project to the electrode fields for correcting their indeterminacies.

#### 2.2.2. Transforming the Back-Projected Components into Time-Frequency Representations

For the back-projected components ${\left(\underline{Z}\right)}^{T}$ from the original signal $\hat{Z}$, we turned this time domain signal to time-frequency domain signal ${\underline{Z}}_{\text{TF}}$ using the complex morlet wavelet transform [20, 38–44]. Specifically, a mother wavelet was first defined using a set of bandwidth and centre frequency. Then, the frequency range of interest (e.g., 0.5-14.5 Hz) and frequency bins were set for calculation of TFR. Next, the baseline correction was finished using the values of each point in the time-frequency distribution subtracting the mean power of the baseline (for instance, 200 ms before the stimulus onset).

#### 2.2.3. Objectively Determining the Region of evoked EROs via Edge Detection Algorithm

The conventional rectangle method was widely used to determine the regions of evoked EROs [5–10, 45]. As the demonstration in section 3.2.1, different statistical results could be displayed because the conventional rectangle method was a subjective method to calculate the region. To address this, an edge detection algorithm, Canny detector [46], was used to objectively distinguish the shape of evoked ERO for each condition from the TFR distribution, which can precisely and objectively mark the position of the oscillatory responses in the TFR based on their shapes (time and frequency positions) [47–49].

**Figure 6:**
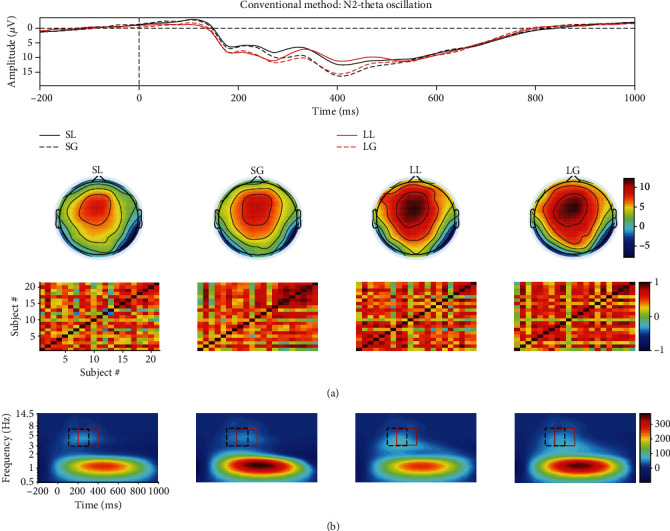
(a) The grand averaged waveform (at Fz, FCz, and Cz electrodes), topography (time window: 180-240 ms), and similarity of topographies across participants of each condition for the filtered real signal. (b) The associated grand averaged time-frequency representation (TFR) of every condition. The region of evoked ERO of each condition is determined by the edge detection algorithm and the conventional rectangle method (for the black dotted rectangle, the time window is 100-300 ms and frequency range is 3-7 Hz, “R1”; the red solid rectangle: 200-400 ms and 3-7 Hz, “R2”) separately. SL: loss condition under short waiting time; SG: gain condition under short waiting time; LL: loss of long waiting time; LG: gain of long waiting time.

**Figure 7:**
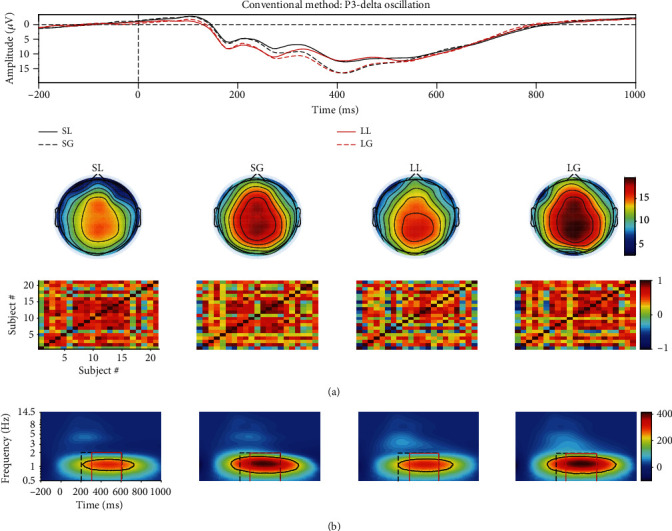
(a) The grand averaged waveform (at Fz, FCz, Cz, CPz, and Pz electrodes), topography (time window: 300-600 ms), and similarity of topographies across participants of each condition for the filtered real signal. (b) The associated grand averaged time-frequency representation (TFR) of every condition obtained by the conventional TFA. The region of evoked ERO of each condition was determined by the edge detection algorithm and the conventional rectangle method separately (for the black dotted rectangle, the time window was set from 200 to 600 ms and frequency range was defined from 0.5 to 2 Hz, “R7”; the red solid rectangle: 300-600 ms and 0.5-2 Hz, “R8”) separately.

**Figure 8:**
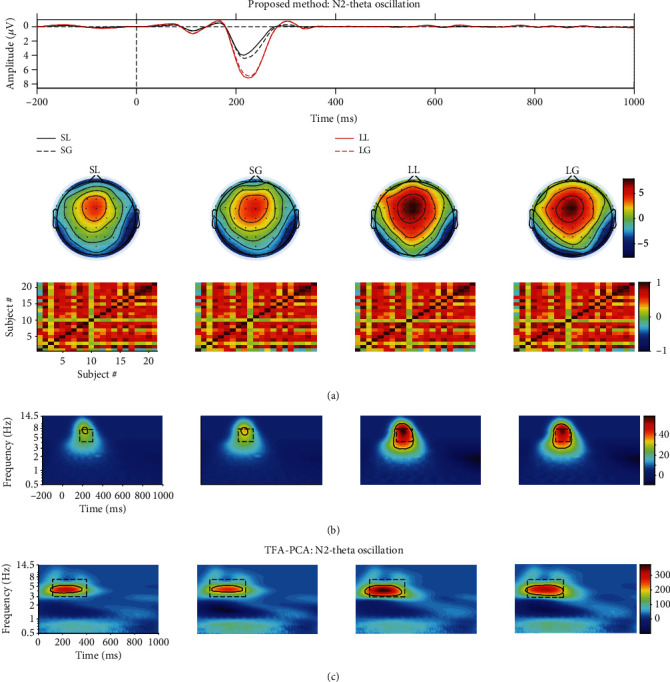
(a) The projected waveform (at Fz, FCz, and Cz electrodes), topography (180-240 ms), and similarity of topographies across participants of every condition for N2 which were extracted from the real mixed signal using t-PCA and Promax rotation. (b) The associated grand averaged time-frequency representation (TFR) of every condition for N2-theta oscillation using the proposed approach. The black dotted rectangle (the time window was defined as 150-300 ms, and the frequency range was set as 4-8 Hz, “R3”) for every condition was marked using the conventional rectangle method, and the other (“R4”) was gained by the edge detection algorithm. (c) The weighted N2-theta oscillation by TFA-PCA. The black dotted rectangle was 100-400 ms and 3-7 Hz (“R5”), and the other one (“R6”) was gained by edge detection algorithm.

**Figure 9:**
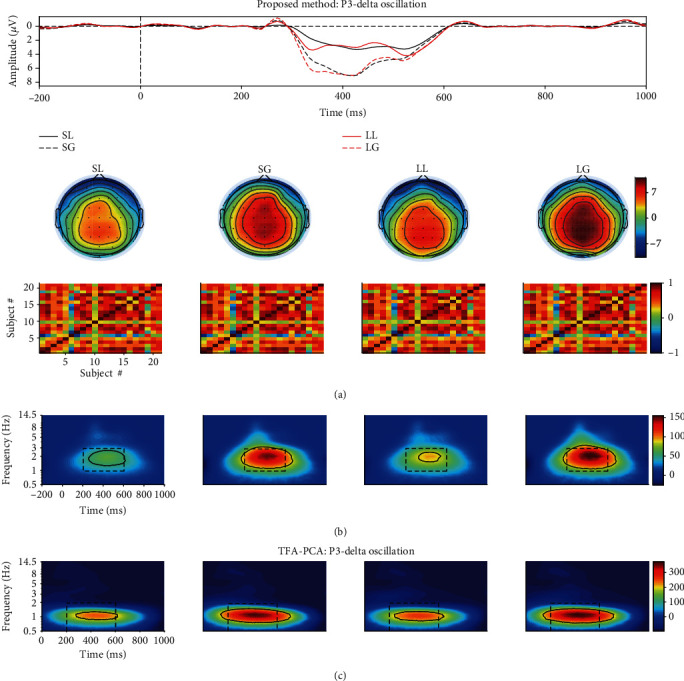
(a) The projected waveform (at Fz, FCz, Cz, CPz, and Pz electrodes), topography (300-600 ms), and similarity of topographies across participants of every condition for N2 which were extracted from the real mixed signal using proposed approach. (b) The associated grand averaged time-frequency representation (TFR) of every condition. The black dotted rectangle (the time window was 200-600 ms, and the frequency range was 1-3 Hz, “R10”) for every condition was marked using the conventional rectangle method, and the other (“R11”) was gained by the edge detection algorithm. (c) The weighted TFRs of P3-delta oscillation when using TFA-PCA. The black dotted rectangle was 200-600 ms and 0.5-2 Hz (“R12”), and the other one (“R13”) was gained by edge detection algorithm.

The displayed TFR was usually generated from ${\underline{Z}}_{\text{TF}}$ by calculating the mean values of the specific electrodes. In this study, we used the symbol *φ*_*f*,*t*,*c*,*s*_ to represent the value of any point in TFR distribution for *s*^th^ subject under *c*^th^ condition. As shown in Figures 6–9, the interested evoked ERO of each condition had a boundary that clearly distinguished evoked ERO from others in the TFR distribution. Following this context, we can use a typical approach, Canny detection algorithm, to determine the optimal boundary and then gain the associated region of evoked ERO.

The procedure of the original Canny algorithm for the determination of the boundary of a target can be approximately divided into the following steps [46, 50].

First, any noise was filtered out from the original image using Gaussian filter before trying to use this detector to detect any edges. Indeed, this step was to calculate the convolution between the raw image and the mask.

Second, aiming to find the edge strength, the gradient amplitude and direction at any pixel location were calculated. The gradient amplitude was determined as the square root of the sum of the square of the horizontal *G*_*x*_(*i*, *j*) and vertical gradient *G*_*y*_(*i*, *j*) amplitudes.

(1) $$\begin{array}{c} G\left(i,j\right)=\sqrt{G_{x}{\left(i,j\right)}^{2}+G_{y}{\left(i,j\right)}^{2}}. \end{array}$$

Then, the gradient direction at every pixel can be defined as follows:

(2) $$\begin{array}{c} \theta_{G}\left(i,j\right)=\text{arctant}\left(\frac{G_{y}\left(i,j\right)}{G_{x}\left(i,j\right)}\right). \end{array}$$

Third, the nonmaxima suppression was applied to the gradient amplitude to make the blurred edges sharper. In other words, the gradient direction at every pixel was computed to find the maximum magnitude. For one thing, when the gradient direction of this pixel was considered as one of 8 possible primary directions (i.e., 0 degree, 45 degrees, 90 degrees, 135 degrees, 180 degrees, 225 degrees, 270 degrees, and 315 degrees), the comparisons were made between the gradient magnitude of this pixel and its two neighbours along the gradient direction. If this value was the greatest one, it was then remained and otherwise, it would be set to zero. For another thing, if the gradient direction was not belonging to any of these possible directions, it would be finished to calculate the neighbouring gradients based on interpolation theory [50].

Fourth, the edge map was determined via hysteresis thresholding. It needed two thresholds to better recognize the edges: a high threshold *T*_1_ and a low one *T*_2_. If the value of any pixel was (i.e., the gradient amplitudes *G*(*i*, *j*)) greater than *T*_1__,_ it would be looked as strong edge and then recorded. Meanwhile, if the gradient amplitudes of the pixels were greater than *T*_2_ and connected to the strong edges, those pixels would be selected as strong edges. Otherwise, they were not included in the final edge image.

Practically, the region of interest needed to be determined based on the recognized boundary for further statistical analysis. Any position (it was determined by a frequency bin- *f*_1_ and a time point - *t*_1_) within the marked boundary was first calculated by performing on the frequency bins, time points, and the pixels of the boundary. Each value *ψ*_*f*,*t*,*c*,*s*_ of the point within the determined boundary was remained for every subject *s* under each condition *c* at electrodes of interest as below.

(3) $$\begin{array}{c} \psi_{f,t,c,s}=\left\{\begin{array}{c} \;\varphi_{f,t,c,s}f=f_{1},\;t=t_{1}\\ 0\text{otherwise} \end{array}.\right. \end{array}$$

Last, the demanded value$\;{\overset{¯}{\psi }}_{c,s}$ for each subject of each condition was gained by computing the mean value of the marked evoked ERO. Note that the parameters of *T*_1_ and *T*_2_ were set with the default values in the Matlab function (version 2018b, the Mathworks, Inc., Natick, MA).

## 3. Results

### 3.1. Synthetic Dataset Results

Figures 5(a)–5(i) show the correlation coefficients between any two waveforms/topographies/TFRs of source, mixed, and projected N2 (theta)/P3 (delta) for the synthetic datasets with different levels of noise, respectively. Meanwhile, Figures 5(j) and 5(k) show the correlation coefficients between the weighted TFR of source N2/P3 and early and late theta oscillations, respectively, for different noise-contaminated simulated datasets using TFA-PCA. Noticeably, all the correlation coefficients between the waveforms/topographies/TFRs of source and extracted N2/P3 for different noise-contaminated simulated datasets were almost equal to 1 (see Figures 5(b), 5(e), and 5(f)), whereas the unstable results were obtained when using TFA-PCA (see Figures 5(j) and 5(k)). Those indicated that evoked ERO for each ERP of interest could be stably and efficiently extracted from low to high SNR-simulated datasets by our proposed approach but not for TFA-PCA approach.

Afterward, we used the results of one simulated dataset (i.e., SNR is 10 dB) to explain the application and assess the performance of the proposed approach and TFA-PCA approach.

In the application of the proposed approach, 17 components were retained, which explained 99% of variance. According to the temporal and spatial properties of P3 and the similarity of the spatial components over all subjects (we used “spatial similarity” to represent it in the following parts), the 1st, 3rd, and 10th components were selected for P3 and they explained 68.02% (spatial similarity: 0.89 ± 0.07), 3.86% (spatial similarity: 0.87 ± 0.07), and 1.39% (spatial similarity: 0.41 ± 0.28) of variance, respectively. Similarly, the 2nd and 5th components were chosen for N2, and they accounted for 6.60% (spatial similarity: 0.73 ± 0.10) and 2.07% (spatial similarity: 0.36 ± 0.19) of variance, respectively.

As shown in Figures 3(b) and 4(b), the power of source N2-theta oscillation (about 0.3 *μ*V^2^/Hz) was much smaller than that of source P3-theta oscillation (approximately 3 *μ*V^2^/Hz) so that the former easily disappeared in the TFR of the mixed signal. This was confirmed in the TFR of the mixed signal, and that is to say, only one oscillation was observed. This was also proved by the correlation coefficient method. Specifically, the correlation coefficient between the TFRs of the mixed and source/extracted N2-theta was roughly 0.74/0.69 while this value was approximately 0.95/0.96 for P3-delta (see Figures 5(g) and 5(i)). The correlation coefficients between the waveforms of the mixed and source/projected N2/P3 were about 0.52/0.95 (see Figures 5(a) and 5(c)). This meant that P3 made the biggest contribution to the mixed signal that led to the abovementioned situation, and consequently, N2 accounted for a small part.

Two evoked EROs were obtained corresponding to N2 (see Figure 3(b)) and P3 (see Figure 4(b)), respectively, when using the proposed approach. What is more, the similarity of topographies across all subjects of the projected signal (especially for N2: from 0.64 ± 0.11 to 0.72 ± 0.09) was improved using the proposed approach when compared with the similarity of the mixed signal. Through the comparisons of the waveforms/topographies/TFRs of the source, mixed, and projected signals as shown in Figures 3 and 4, we could easily obtain that they were almost identical with each other, respectively. Regarding the correlation coefficients between the waveforms/topographies/TFRs of the source and projected signals for N2-theta/P3-delta, obviously, they were all roughly equal to 1.00 (see Figures 5(b), 5(e), and 5(h)). Hence, we concluded that the proposed approach can efficiently and objectively extract the ERPs of interest from the mixed signals.

With regard to the results of TFA-PCA, 7 components were retained, which was explained 99% of variance. Then, the 2nd and 3rd components were, respectively, weighted with the original TFRs and the weighted results, respectively, corresponded to late and early theta oscillations. They were just classified as one part of the theta oscillation of TFRs for the mixed signal (Figure 4(c)) due to their time window and frequency range were similar with the original theta oscillation. This was demonstrated by the correlation coefficients (0.79/0.59 and 0.51/0.57) between the TFRs of the weighted early/late theta oscillations and the source N2/P3 separately (see Figures 5(j) and 5(k)).

### 3.2. Actual ERP Dataset Results

#### 3.2.1. Conventional Time-Frequency Analysis Results

For N2-theta oscillation in Figure 6(b), the statistical results of the two regions determined by the conventional rectangle method demonstrated that no significant differences were found for either the main effect of feedback or interaction effect as shown in “R1” and “R2” of Table 1, whereas the main effect of waiting time reached significant level. The related region for LL condition was not recognized when we used the edge detection algorithm, and thus, the statistical analysis was not further processed.

As for the P3-delta oscillation in Figure 7(b), the statistical results of the determined regions obtained by the conventional rectangle method indicated that the main effect of feedback was significant but not for the waiting time. In addition, the interaction effect between waiting time and feedback was also insignificant (see Table 2, “R7” and “R8”). However, we did not find any significant main or interaction effects for the ANOVA results when using the edge detection algorithm (Table 2, “R9”).

#### 3.2.2. Proposed Approach Results

Figures 8 and 9 depict the projected waveform at some typical electrodes, the topographic distribution in the time domain, associated similarity of topographies across all subjects, and TFR of every condition for N2-theta and P3-delta, respectively. 20 components were retained, and they accounted for 99% of the variance when applying t-PCA and Promax rotation.

The 9th and 18th components were finally selected for further analysis based on the properties of N2 in the temporal and spatial and the similarity of spatial components across all subjects (we used “spatial similarity” to represent it in the following parts), and they explained 0.91% (spatial similarity: 0.44 ± 0.30) and 0.15% (spatial similarity: 0.59 ± 0.28) of variance, respectively. The evolution and the tendency of the projected N2 waveform kept consistent with the conventional grand averaged waveform. For the recognized regions of the evoked theta for TFRs of the projected N2 by the edge detection method (Table 1, “R4”), the related statistical results indicated that the main effect was insignificant for either waiting time (*F*_(1, 20)_ = 3.122, *p* = 0.093, and *η*_*p*_^2^ = 0.135) or feedback (*F*_(1, 20)_ = 0.382, *p* = 0.543, and *η*_*p*_^2^ = 0.019). Meanwhile, the interaction effect between waiting time and feedback was also not significant (*F*_(1, 20)_ = 0.633, *p* = 0.436, and *η*_*p*_^2^ = 0.031). These findings were consistent with the previous study of the results for the time domain analysis [18]. Nevertheless, when the conventional rectangle method was performed to determine the region (“R3”: the time window is 150-300; the frequency range is 4-8 Hz) for TFR of each condition, we found a significant main effect of waiting time factor (*F*_(1, 20)_ = 8.92, *p* = 0.009, and  *η*_*p*_^2^ = 0.298), whereas the other main or interaction effect did not reach a significant level.

Similarly, with regard to P3, the 1st, 5th, 13th, 14th, 16th, and 17th components (they explained 52.7% (spatial similarity: 0.66 ± 0.20), 3.82% (spatial similarity: 0.46 ± 0.33), 0.31% (spatial similarity: 0.58 ± 0.25), 0.27% (spatial similarity: 0.66 ± 0.20), 0.19% (spatial similarity: 0.70 ± 0.17), and 0.16% (spatial similarity: 0.54 ± 0.32) of the variance, respectively) were selected and projected back to the electrode fields. We then computed the TFRs of the back-projection via wavelet transform. The results revealed that the long waiting time (96.583 ± 21.773 *μ*V^2^/Hz) elicited a larger power than short waiting time (76.251 ± 18.461 *μ*V^2^/Hz). A larger power was also observed upon gain condition (106.238 ± 26.993 *μ*V^2^/Hz) than lose condition (66.596 ± 13.773 *μ*V^2^/Hz), which was similar with the previous investigations [51–53]. The statistical results of the determined regions obtained by the edge detection algorithm (Table 2, “R11”) displayed that there was a significant interaction effect between waiting time and feedback (*F*_(1, 20)_ = 9.573, *p* = 0.006, and *η*_*p*_^2^ = 0.324). However, this significant interaction effect between them was not found in the previous study [18]. In addition, the main effects of both waiting time (*F*_(1, 20)_ = 6.886, *p* = 0.016, and *η*_*p*_^2^ = 0.256) and feedback (*F*_(1, 20)_ = 5.886, *p* = 0.025, and *η*_*p*_^2^ = 0.227) reached a significant level. Then, post hoc analysis was used for further investigation. The results demonstrated that a significant difference was found in the feedback factor under short waiting time condition (*p* = 0.007). By contrast, there was an insignificant main effect of feedback under long waiting time condition (*p* = 0.172). However, when the rectangle method was applied to determine the region (time window is from 200 to 600 ms, and the frequency range is 1-3 Hz), only the significant main effect was observed for feedback factor (*F*_(1, 20)_ = 7.755, *p* = 0.011, and *η*_*p*_^2^ = 0.279).

#### 3.2.3. TFA-PCA Results

Eight components were reserved when TFA-PCA was performed on the TFRs of the averaged ERP waveforms, which explained 99% of the total variances. The 1st (75.15%) and 4th (1.76%) were selected to weight with the original TFR, and the weighted TFRs were associated with delta and theta oscillation, respectively, as depicted in Figures 8(c) and 9(c). The statistical results of all determined delta/theta oscillation obtained by the conventional rectangle method and edge detection algorithm revealed that either main or interaction effects did not reach a significant level (see the statistical results of “M3” in Tables 1 and 2).

## 4. Conclusion and Discussion

We developed a novel approach to objectively explore evoked event-related oscillations (EROs) of interest mainly including three steps: (1) temporal principal component analysis (t-PCA) and Promax rotation were performed on the ERP waveform matrix to extract the temporal and spatial components of interest simultaneously and then the components of interest were projected to the electrode fields to correct their indeterminacies in the variance and the polarity. (2) The time-frequency representations (TFRs) of the back-projection waveforms were computed using the complex morlet continuous wavelet transform in the electrode fields. (3) The edge detection algorithm based on the Canny detector was applied on the TFRs to recognize the specific time and frequency positions of evoked EROs at some typical electrodes for the further statistical analysis.

As displayed in Figures 5(b), 5(e), and 5(f), all the correlation coefficients between the waveforms/topographies/TFRs of source and extracted N2/P3 for different noise-contaminated simulated datasets were roughly 1. However, the correlation coefficients between TFRs of weighted and source N2/P3 signals were easily influenced by noise using TFA-PCA (see Figures 5(j) and 5(k)). These mean that our proposed approach could efficiently extract evoked EROs of interest from a series of SNR signals but not for TFA-PCA approach. Hereafter, we use the results of one noise-polluted simulation data (i.e., 10 dB) to explain that the proposed approach outperformed and TFA-PCA. Specifically, only one identifiable delta oscillation around 300-600 ms can be recognized from the TFR distribution for the mixed synthetic dataset by wavelet transform method as shown in the second row in Figures 3(b) and 4(b). In contrast, two oscillations were obtained by the proposed approach which corresponded to N2 and P3, respectively, as illustrated in Figures 3(b) and 4(b). Early and late theta oscillations were gained by TFA-PCA, but we categorized them as one part of the theta oscillation of the mixed signals. Likewise, the statistical results of the real dataset for the proposed approach were more satisfied with experimental purpose than TFA and TFA-PCA approaches as follows. P3-delta statistical results of the proposed approach revealed that the loss condition reflected smaller power than the gain did, which was similar to the results in the previous reports [51–54]. Besides, the interaction effect between the two factors was significant, which was consistent with the findings in the prior study [51]. However, when we applied the conventional TFA method to the signals, only the distinction between the two feedback conditions was found for delta oscillation. Meanwhile, we did not find any significant main or interaction effect when using the TFA-PCA approach.

The proposed approach is an efficient and objective method. Firstly, the results of three applied methods, which were described in Tables 1 and 2, revealed that the statistical results achieved by the edge detection algorithm were more stable when compared with those of the conventional rectangle method [5–10, 55]. Secondly, the statistical results of P3-delta for the conventional TFA/TFA-PCA and the rectangle method did not reflect that the feedback was sensitive to the waiting time probably due to the following aspects. (a) The evoked P3-delta was overlapped with other EROs and artifacts to some degree in the temporal, spatial, and spectrum. (b) Information of each ERO for the projected signal cannot be completely included or some useless information was involved when using the rectangle method to determine the region of interest, such as the region was marked as shown in Figures 8(b) and 9(b). However, when using our approach, we found that the power values of feedback for the long and short waiting time were significantly different, which was consistent with the previous study [51].

Furthermore, Promax rotation is used to rearrange the initial principal components (PCs) such that PCs have a simple and more interpretable structure in the time domain. We expect that one PC can interpret one ERP but the generated PC will not have a simple relationship with ERP, for example, one PC might be a part of the P2 plus a part of N2 and plus a part of P3 and so forth. Several rotation approaches have been developed for this purpose, and the key difference between them is whether they are oblique or orthogonal, that is, whether the PCs are forced to be correlated or not. Varimax and Promax are the typical algorithms for orthogonal and oblique rotations, respectively, which the former forces the PCs to be uncorrelated while the latter allows the PCs to be related. The previous study revealed that Promax rotation can yield much better results than Varimax rotation both in real and simulated ERP datasets [31, 56–58], and Promax rotation can give the improved results for t-PCA [32, 56]. Therefore, we applied Promax rotation during t-PCA procedure to rotate the original extracted PCs in this study.

Moreover, the selection of the components in this study also depends on the similarity of the topographies of different subjects, and it is expected that different subjects' topographies are as homogeneous as possible. Regarding one component of the t-PCA plus Promax rotation, all subjects in one group have the same temporal course and variant spatial components (i.e., topographies here). This means that, for t-PCA and Promax rotation, given an estimated ERP component, the waveform is invariant for all subjects and its topography is variant across all subjects. However, it is strongly expected that the topographies across different subjects for an ERP can be as similar as possible since we expect a homogenous ERP dataset for the repeatable and reliable data analysis. For the results of synthetic and real datasets, the similarities were improved for the projected components to some extent after the proposed method was used (especially for N2 of the extracted signal (0.72 ± 0.09) when compared with the mixed signal (0.64 ± 0.11) as illustrated in the last column the Figure 3(a)). This demonstrated that the homogeneity of the topographies of different subjects was better than before with the proposed approach.

Another technique can also be used to identify the region of evoked ERO of interest based on the subtle change for their topographies as used in a previous study [59]. In order to identify the precise region (i.e., uniquely topographic was included in the identified region) for the ERO of interest, two main stages were involved as below [59]. First, TFRs were obtained from either averaged or sing-trial ERP signals. Second, all time-frequency points were divided into time-frequency features (i.e., regions) based on the correlation coefficients of topographies between the time-frequency points and templates using k-means cluster. Likewise, in this study, we used the following steps to gain the “pure” regions for evoked EROs of interest. The components of interest corresponded to the EROs of interest were first extracted from the averaged ERP dataset in the time domain using t-PCA and Promax rotation. Next, we calculated the TFRs of the extracted signals and identified the regions of evoked EROs of interest using the edge detection algorithm. Obviously, the former approach can be used to explore the EROs from both averaged and single-trial ERP datasets, but the proposed approach only extracts the ERO from the averaged ERP datasets. Furthermore, it should be noted that if the edge detection algorithm is directly used to recognize the regions of EROs from the TFRs of the original averaged ERP data, consequently, different spatially distinct oscillations may be involved in one region when those components are overlapped in time and frequency domains. By contrast, this situation will not happen for the results obtained by topographic segmentation analysis. That is, the spatial distributions of all points in the same region are highly similar to each other [59].

There are some potential drawbacks to the proposed approach. Firstly, only the time-locked and phase-locked information of the event-related responses can be explored due to we first performed our proposed approach on the averaged ERP datasets to extract components of interest and then calculated the related TFRs to find the time-frequency features. Secondly, the selection of the temporal and spatial components obtained by t-PCA and Promax rotation might be affected by the experimenters. Although we give a criterion that the extracted components are chosen for further analysis when the properties of components in the time and space domains are consistent with ERPs, the experimenters can still determine which component was involved in the next stage. Thirdly, we have to define a mother wavelet by a set of bandwidth and frequency centre (BWCF) before we used morlet wavelet transformation to transform the time-domain signals into time-frequency signals. According to our previous study [20], different sets of BWCF could lead to different time-frequency results; thus, the experimenters have to attempt the number of BWCF for TFA and then select an optimal one from them for the TFA of ERP signals.

Regarding the future investigations, it can be carried out from the following aspects. Firstly, we merely focused on the extraction of evoked oscillations from the averaged ERP as mentioned above in this study. It should be noted that some important information like induced oscillation was cancelled out by the averaging procedure over trials in the time domain [60, 61]. In addition, the induced oscillatory response was probably generated by nonlinear and possibly autonomous mechanisms, and it would belong to high-order processes. Whereas evoked oscillation was related to stimulus-locked time [17]. In past decades, the induced oscillation had been widely used to investigate the neural mechanisms of attention modulation [62], the functions of the alcohol use disorder patients [63], and so on. Our proposed approach used for the single-trial level analysis will be helpful to explore the mechanisms of the induced oscillation in the mentioned fields. Secondly, regarding the selection of components of interest from the extracted ones obtained by t-PCA and Promax rotation, one strategy can be used based on the absolute of the correlation coefficients between any two extracted spatial components and the peak time points for the extracted component. For example, there are two extracted components, their spatial correlation coefficient is 0.9 and the peak time points are 190 ms and 220 ms. As a result, they are considered as one thing and are projected together onto electrode fields for further analysis. Thirdly, some TFA techniques with free parameter settings, like the combination of Wigner-Ville distribution and Gabor transform with the matching pursuit decomposition, can provide an appropriate time-frequency resolution in all frequencies [64], which can also be applied to the ERO analysis as the alternatives to the proposed approach in our study.

## Figures and Tables

**Table 1:** The statistical results of N2-theta oscillation for the conventional time-frequency analysis (“M1”), the proposed approach (“M2”), and TFA-PCA (“M3”).

Method	ROI		WT			FB			WT∗FB	
		*F*	*η*_*p*_^2^	*p*	*F*	*η*_*p*_^2^	*p*	*F*	*η*_*p*_^2^	*p*
M1	R1	6.067	0.233	0.023	0.72	0.035	0.406	0.206	0.01	0.655
	R2	4.853	0.195	0.039	0.042	0.002	0.84	0.038	0.002	0.848
M2	R3	8.492	0.298	0.009	0.052	0.003	0.821	0.569	0.028	0.46
	R4	3.122	0.135	0.093	0.382	0.019	0.543	0.633	0.031	0.436
M3	R5	3.255	0.14	0.086	0.084	0.004	0.775	0.006	<0.001	0.941
	R6	0.393	0.019	0.0538	0.411	0.02	0.529	0.015	0.001	0.903

R1: 100-300ms and 3-7 Hz; R2: 200-400 ms and 3-7 Hz; R3: 150-300 ms and 4-8 Hz; R4: EDM; R5: 100-400 ms and 3-7 Hz; R6: EDM. EDM: the edge detection method; WT: waiting time; FB: feedback; ROI: region of interest.

**Table 2:** The statistical results of P3-delta oscillation for the conventional time-frequency analysis (“M1”), the proposed approach (“M2”), and TFA-PCA(“M3”).

Method	ROI		WT			FB			WT∗FB	
		*F*	*η*_*p*_^2^	*p*	*F*	*η*_*p*_^2^	*p*	*F*	*η*_*p*_^2^	*p*
M1	R7	0.997	0.048	0.33	13.236	0.398	0.002	0.25	0.012	0.622
	R8	1.027	0.049	0.323	12.653	0.387	0.002	0.167	0.008	0.688
	R9	0.064	0.003	0.802	2.634	0.116	0.12	0.004	<0.001	0.952
M2	R10	3.93	0.164	0.061	7.755	0.279	0.011	2.991	0.13	0.099
	R11	6.886	0.256	0.016	5.886	0.227	0.025	9.573	0.324	0.006
M3	R12	1.007	0.048	0.328	12.299	0.381	0.002	0.274	0.014	0.607
	R13	0.125	0.006	0.727	2.141	0.097	0.159	0.06	0.003	0.809

R7: 200-600ms and 0.5-2 Hz; R8: 300-600 ms and 0.5-2 Hz; R9: EMD; R10: 200-600 ms and 1-3 Hz; R11: EDM; R12: 200-600 ms and 0.5-2 Hz; R13: EDM. EDM: the edge detection method; WT: waiting time; FB: feedback; ROI: region of interest.

## Data Availability

The datasets used for supporting the findings in this study are available from the corresponding author on reasonable request.

## Appendix

### A. The Explanation of Temporal PCA and Promax Rotation from the View of Blind Source Separation

When applying temporal PCA and Promax rotation [65] to decompose an ERP dataset **Z**^*T*^ ∈ *ℛ*^*N*×*M*^ (*N* and *M*, respectively, represent the number of sensors of all subjects under all conditions and the number of time points within one epoch), the related procedures can be interpreted via the linear model as below [34, 35].

(4) $$\begin{array}{c} Z=HS_{1}+E=H\left(S_{1}+S_{2}\right)=HS, \end{array}$$

where **H** is the mixing matrix with full rank; **S** = **S**_1_ + **S**_2_ (**S** ∈ *ℛ*^*R*×*N*^), **E** = **H****S**_2_, and they are the unknown correlated source signals and the sensor noise, respectively.

As described in [34], the assumption of the model in Eq. (4) is overdetermined and it means that the number of the observed signals *M* is larger than that of the source signals *R*. Once the estimation of the number of the sources is done (the determination can be based on the model order selection algorithms, and here, it is the cumulative explained variance as mentioned in Section 2.2.1 in this study), the overdetermined model can be changed to the determined one as follows

(5) $$\begin{array}{c} X=V^{T}Z=V^{T}HS=AS, \end{array}$$

where **X** ∈ *ℛ*^*R*×*N*^; **V**^**T**^ ∈ *ℛ*^*R*×*M*^ represents the dimension reduction matrix generated from performing PCA on **Z**^*T*^, **A** ∈ *ℛ*^(*R* × *R*)^ is also named the mixing matrix.

Aiming to separate the mixture in Eq. (5), the blind source separation algorithm can be applied [33]. Here, Promax rotation [65] is used to obtain an unmixing matrix **W** in this study. The algorithm of Promax rotation is described in *Appendix B* and the inverse matrix **B** = **W**^−1^ represents the estimation of **A** [34].

And then we use this unmixing matrix **W** to turn the mixture in Eq. (5) into a matrix of estimated components as below [34].

(6) $$\begin{array}{c} Y=WX=\text{WAS}=CS. \end{array}$$

In the above formulation, **Y** is the estimation of **S** and its each row can be assembled to the topographic map of each source; **C** = **W****A** is the global matrix.

Under this determined model condition in Eq. (5), aiming to solve the issue that different components, which are derived from the matrix **X**, cannot be statistically further analysed together because their polarities and amplitudes are indeterminates [33], and we project the component(s) of interest back to the electrode fields in this study which is always used in many other blind source separation for EEG, for example, independent component analysis procedures [34–37]. The back-projection can be illustrated as [34–36].

(7) $$\begin{array}{c} Q_{r}=b_{r}\circ y_{r}, \end{array}$$

here, **Q**_**r**_ ∈ *ℛ*^*R*×*N*^ is the projection of *r*^th^ component at all virtue time points in this study; **b**_*r*_ is the *r*^th^ column of the inverse matrix **B**, and **y**_*r*_ is the *r*^th^ row of the estimated matrix **Y**; “∘” denotes the outer product of vectors.

Under the global optimization, there exists only one nonzero element in each row and each column of **C**. In other words, the *r*^th^ extracted component is unknown scaled version of the *j*^th^ source signal. Hence, the projection in Eq. (7) can be described as [34, 35].

(8) $$\begin{array}{c} Q_{r}=b_{r}\circ y_{r}=a_{j}\circ s_{j}, \end{array}$$

where **a**_*j*_ is the *j*^th^ column of the mixing matrix **A**, and **s**_*j*_ is the *j*^th^ row of the source matrix **S** in Eq. (5).

To the original overdetermined model in Eq. (4), the *r*^th^ component derived from the matrix **Z** can be projected to the all electrodes as below

(9) $$\begin{array}{c} {\underline{Z}}_{r}=u_{r}\circ y_{r},\\ U=VB, \end{array}$$

where **u**_*r*_ is the *r*^th^ column of **U** and denotes the time course or waveform of multisubject of multicondition. We use combination of the inverse matrix **B** and the dimension reduction matrix **V** to represent **U**, which is achieved to estimate the mixing matrix **H** in Eq. (4) [34]. This has been illustrated by Figures 3 and 4.

In most cases, several components need to be projected back to electrodes simultaneously [35, 66]; hence, the related projection of several components can be implemented as follows

(10) $$\begin{array}{c} \underline{Z}=\left[u_{k_{1}},\cdots ,u_{k_{r}}\right]\left[y_{k_{1}},\cdots ,y_{k_{r}}\right]=u_{k_{1}}\circ y_{k_{1}}+\cdots +u_{k_{r}}\circ y_{k_{r}}, \end{array}$$

where *k*_1_, ⋯, *k*_*r*_ (1 ≤ *k*_*r*_ < *R*) are the number of selected components; “∘” denotes the outer product of vectors. The size of each dimension of the matrix $\underline{Z}\;$is the same with that of **Z**. This has been illustrated by Figures 3, 4, 8, and 9.

### B. Oblique Procrustes Transformation

Mathematically, Procrustes equation can be defined as [65, 67].

(11) $$\begin{array}{c} P=VW+\underline{E}, \end{array}$$

where **P** is called as the pattern matrix; **W** is the transformation matrix; $\underline{E}$ is the residual matrix. The satisfactory result is that we can find a transformation matrix to make the value of ${\underline{E}}^{T}\underline{E}$ as close zero as possible.

Specifically, the pattern matrix **P** is first generated from the matrix of unrotated factor loadings **V** by the target Procrustes transformation, and the determination of **V** is based on PCA here, and it is used in the Eq. (5).

(12) $$\begin{array}{c} p\left(i,j\right)=\frac{\left|\text{v}{\left(i,j\right)}^{k+1}\right|}{\text{v}\left(i,j\right)}, \end{array}$$

where *k* > 1. The matrix **P** stands for the matrix **V** raised to the *k*^th^ power, and its original sign is unchanged.

Next, the least squares method is performed to calculate the fit of the orthogonal matrix **V** of the factor loadings to the pattern matrix so that ${\underline{E}}^{T}\underline{E}$ is a minimum.

(13) $$\begin{array}{c} W={\left(V^{T}V\right)}^{-1}V^{T}P, \end{array}$$

where **W** is called the transformation matrix in Promax rotation [67]; **V**^**T**^ is the transpose matrix of the orthogonal rotated matrix **V**; (^.^)^−1^ is the inverse of a matrix.
